# The targeting imaging and treatment capacity of gelsolin-targeted and paclitaxel-loaded PLGA nanoparticles *in vitro* and *in vivo*

**DOI:** 10.3389/fbioe.2022.933856

**Published:** 2022-10-20

**Authors:** Jiamei Ji, Haocheng Qin, Yan Yang, Jun Wu, Juan Wu

**Affiliations:** ^1^ Department of Ultrasound, The Second Affiliated Hospital of Dalian Medical University, Dalian, Liaoning, China; ^2^ Department of Ultrasound, Lianyungang First People’s Hospital, Lianyungang, Jiangsu, China; ^3^ Department of Ultrasound, Huainan First People’s Hospital, Huainan, Anhui, China; ^4^ Department of Ultrasound, Lanzhou University Second Hospital, Lanzhou, Gansu, China

**Keywords:** targeted therapy, gelsolin, paclitaxel, lymphatic metastasis, ultrasound imaging

## Abstract

As a vital sign of carcinomas, lymph node metastasis is closely related to poor prognosis due to a lack of identification and effective treatment in the early stage. Nanoscale contrast agents targeting specific tumor antigens are expected to identify tumor metastasis in the early stage and achieve precise treatment. As a biomarker in the early stage of tumor invasion and metastasis, gelsolin (GSN) might be a promising molecular target to identify and screen tumor metastasis through the lymphatic system. Therefore, GSN-targeted paclitaxel-loaded poly(lactic-co-glycolic acid) nanoparticles (GSN-PTX-PLGA NPs) were prepared, and their physicochemical properties, encapsulation efficiency, drug loading, and dissolution were determined. Besides, uptake experiments and the fluorescent imaging system were used to evaluate their targeting capability. The targeting imaging and treatment capacity were also assessed by experiments *in vitro* and *in vivo*. The diameter of the GSN-PTX-PLGA NPs was 328.59 ± 3.82 nm. Hca-F cells with GSN-PLGA NPs showed stronger green fluorescence than Hca-P cells. DiI-labeled GSN-PLGA NPs in tumor-bearing mice and isolated organs exhibited more prominent fluorescence aggregation. The imaging of GSN-PLGA NPs was satisfactory *in vitro*, and the echo intensity gradually increased with increasing concentrations of GSN-PLGA NPs. After treatment with GSN-PTX-PLGA NPs, there was an obvious decrease in tumor volume and lymph node metastasis rate compared to the other groups (*p* < 0.05). In conclusion, GSN-PTX-PLGA NPs have a remarkable targeting capacity *in vivo* and *in vitro,* and they effectively inhibit tumor growth and lymph node metastasis *in vivo*.

## Introduction

As a vital sign of carcinomas, lymph node metastasis is closely related to poor prognosis due to a lack of identification and effective treatment in the early stage (Ji, 2016; Garnier et al., 2019). The characteristics of tumor morphology and perfusion pattern were clinically dominant imaging diagnostic basis by far. The use of ultrasound contrast agents enhances and reveals the characteristics of organ perfusion in space and time, contributing to the differential diagnosis of benign and malignant tumors to some extent (De Leon et al., 2018). However, the particle size of traditional contrast agents is usually larger than 1 μm, which prevents tissue space penetration, resulting in the inability to detect the biological behaviors of tumors (Han et al., 2022). Therefore, nanoscale contrast agents targeting specific tumor antigens or loading therapeutic agents were widely explored and expected to identify benign and malignant tumors and allow precise treatment (Rapoport et al., 2011; Karimi et al., 2017). In the aspect of targeting agents, most investigations were focused on tumor angiogenesis and metabolism. However, studies on identifying lymph node metastasis by targeting nanoparticles (NPs) were still limited.

Gelsolin (GSN), an actin-binding protein, plays a crucial role in lymph node metastasis of tumors (Wu et al., 2013; Huang et al., 2022). GSN is highly expressed in a series of human cancers, such as bladder cancer and hepatocarcinoma (Wang et al., 2014; Yang et al., 2020). The Hca-F and Hca-P cell lines are a pair of syngeneic murine hepatocarcinoma ascites cell lines with different lymphatic metastasis potentials. GSN is usually expressed in the cytoplasm and cytoskeleton of Hca-P cells with low lymphatic metastasis potential. It is also expressed on the membranes of Hca-F cells with high lymphatic metastasis potential (Qazi et al., 2011). Therefore, GSN is considered a promising biomarker in the early stage of tumor invasion and metastasis and might become an effective molecular target to identify and screen tumor metastasis through the lymphatic system (Wang et al., 2014; Huang et al., 2022). Recent studies have shown that GSN-targeted ultrasound contrast agents could bind with Hca-F cells due to their high expression of GSN and display a good quality of imaging and therapeutic effects (He et al., 2020; Qin et al., 2022).

Paclitaxel (PTX), a common chemotherapy drug, inhibits the mitosis and proliferation of tumor cells (Zhao et al., 2022). However, the clinical efficacy of PTX is often compromised due to its poor aqueous solubility, low drug utilization rate, and serious adverse effects (Singh and Dash, 2009). Poly(lactic-co-glycolic acid) (PLGA) is a new nanoscale biomaterial and is widely applied to the preparation of contrast agents (Zhou et al., 2021). Due to its favorable stability, biocompatibility, and biodegradability, PLGA is used to prepare nanoparticle shells coupled with active substances, including monoclonal antibodies, ligands, and various specific short peptides, and load drugs on its surface (Mahapatro and Singh, 2011; Wang et al., 2021). These NPs can penetrate the gaps between capillary endothelial cells and disperse into the intercellular spaces to detect and interfere with the biological behaviors of tumors (Xu et al., 2010).

In the present study, we prepared GSN-targeted paclitaxel-loaded PLGA nanoparticles (GSN-PTX-PLGA NPs) and investigated the targeting imaging and therapeutic effects of GSN-PTX-PLGA NPs *in vitro* and *in vivo* to provide new molecular imaging and targeted therapy for lymphatic metastasis of tumors.

## Material and methods

### Materials

A total of 615 female mice aged 4–6 weeks and weighing 20 ± 2 g were obtained from the Experimental Animal Center of Dalian Medical University (Dalian, China). Hca-F and Hca-P cells were provided by the Pathology Department of Dalian Medical University (Dalian, China). The RPMI-1640 Dulbecco’s modified Eagle’s medium (DMEM) and penicillin/streptomycin solution were obtained from HyClone Laboratory (United States). Fetal bovine serum (FBS) was purchased from Biowest Company (France). Poly(lactic-co-glycolic acid)-COOH (PLGA, with a polymerization ratio of 50:50 and molecular weight of 12,000), polyvinyl alcohol (PVA), 1-ethyl-3 (3 dimethylaminopropyl) carbodiimide (EDC), N-hydroxysuccinimide (NHS), GSN antibody, 1,1-dioctadecyl-3,3,3,3-tetramethylindocarbocyanine perchlorate (DiI), 4,6- diamino-2-phenylindole (DAPI), coumarin 6, and Tween 80 were purchased from Sigma Chemical Co., (St. Louis, MO, United States). Methylene chloride and isopropanol were purchased from Chongqing Chuandong Chemical Co., Ltd. (Chongqing, China). PTX was purchased from Beijing Yihe Biological Engineering Co., Ltd. (Beijing, China).

### Preparation of poly(lactic-co-glycolic acid) nanoparticles

Hollow PLGA NPs were prepared by the modified double-emulsion solvent evaporation method. For the first emulsion, 50 mg of PLGA was dissolved in 1 ml of methylene chloride. Subsequently, 0.2 ml of double-distilled water was added, and the polymer solution was sonicated by a VCY-500 sonicator at 100 W for 120 s with alternating 4 s on and 2 s off. For the second emulsion, the emulsified solution was added to 5 ml of 4% PVA and sonicated at 75 W for 90 s with alternating 4 s on and 2 s off, alternately. After the double emulsion, 20 ml of isopropyl alcohol solution (2%, w/v) was added and continuously stirred at room temperature for 2–4 h to evaporate methylene chloride. After evaporation, the samples were collected by centrifugation (8,000 rpm/min for 1 min) with double-distilled water.

### Preparation of gelsolin-targeted paclitaxel-loaded poly(lactic-co-glycolic acid) nanoparticles

PTX-PLGA NPs were prepared by mixing 5 mg of PTX with 50 mg of PLGA. The prepared PTX-PLGA NPs were dissolved in 2-(N-morpholino)ethanesulfonic acid hydrate MES buffer (0.1 mol/L, pH = 5.5), and then EDC/NHS (2:1) was added to the PTX-PLGA NPs. The mixed solution was agitated and incubated for 1 h at room temperature. The remaining EDC/NHS was removed by centrifugation at 8,000 rpm. The activated PLGA NPs were dissolved in MES buffer (pH = 8.0) with a final concentration of 1.5 mmol/L, and 40 μl of GSN monoclonal antibody (200 μg/ml) was added. After incubation at room temperature for 1–2 h, the NPs were centrifuged with double-distilled water at 8,000 rpm/min, and the microspheres were collected.

### Preparation of fluorescence-labeled poly(lactic-co-glycolic acid) and gelsolin-targeted paclitaxel-loaded poly(lactic-co-glycolic acid) nanoparticles

The product was prepared by the double emulsion method in the dark. PLGA (100 mg), a moderate amount of oleic acid NPs, and the DiI fluorescent dye were added to 2 ml of dichloromethane. The mixture was stirred well until it was completely dissolved, and 0.2 ml of double distilled water was then added to the mixture and vibrated for 40 s to form a pink emulsion (W/O microspheres). The emulsion was poured into a 5% PVA solution and homogenized for 5 min to form W/O/W microspheres. Then, 10 ml of the 2% isopropanol solution was added, followed by several washes with double distilled water and centrifugation at 5,000 rpm/min for 5 min to obtain DiI PLGA microspheres.

### Physicochemical properties of gelsolin-targeted paclitaxel-loaded poly(lactic-co-glycolic acid) nanoparticles

The morphology and distribution of GSN-PTX-PLGA NPs were observed under a light microscope (Olympus; Japan) and scanning electron microscope (JEOL, Japan). A laser particle size analyzer (Zeta SIZER, Malvern, United States) was used to obtain the mean diameter and zeta potential.

### Drug-loading and encapsulation efficiency of the gelsolin-targeted paclitaxel-loaded poly(lactic-co-glycolic acid) nanoparticles

A high-performance liquid chromatography (HPLC) method was used to determine drug-loading and encapsulation efficiencies. The amount of PTX recovered from the washed supernatants during preparation was determined. The drug-loading and encapsulation efficiencies were calculated by the following formulas: loading efficiency = W_1_/W_2_ × 100% and encapsulation efficiency = W_1_/W_3_ × 100%, where W_1_ represents the total amount of drug in GSN-PTX-PLGA NPs, W_2_ represents the total weight of PLGA-COOH used for GSN-PTX-PLGA NPs, and W_3_ represents the total weight of PTX used in the preparation of the GSN-PTX-PLGA NPs (Li et al., 2012; Ting et al., 2012). Each process was performed in triplicate.

### Paclitaxel release experiment *in vitro*

GSN-PTX-PLGA NPs were dissolved in PBS (2 ml, pH = 7.4) and transferred to dialysis bags (molecular weight: 8,000 Da), which were placed in a bottle containing 150 ml of slow-release medium and centrifuged at 120 rpm/min at 37°C. At appropriate intervals, 1 ml of the dialysate was removed from the sample, and 1 ml of fresh PBS was added to the sample to maintain the volume. After filtration through a 0.22 μm polyvinylidene fluoride (PVDF), fresh PBS was added. Finally, 20 μl of the sample was collected to calculate the cumulative release rate based on the HPLC method, and the release time curve was traced.

### Attachment of gelsolin monoclonal antibody to the surface of gelsolin-targeted paclitaxel-loaded poly(lactic-co-glycolic acid) nanoparticles

In brief, 10 mg of DiI-labeled PTX-PLGA-GSN NPs and PLGA NPs were fully dissolved in 5 ml of PBS, followed by the addition of 20 μl of FITC-labeled goat anti-mouse IgG antibody, and the mixture was shaken at 4°C for 2 h. The mixture was repeatedly washed with PBS and centrifuged. The attachment of the monoclonal antibody to the surface of GSN-PTX-PLGA NPs was observed under a laser confocal scanning microscope, and the antibody-binding rate was detected by flow cytometry.

## Detection and evaluation of GLN-mediated targets *in vitro*

### Cell culture

Hca-F and Hca-P cells were cultured in RPMI-1640 Dulbecco’s modified Eagle’s medium (DMEM) supplemented with 10% fetal bovine serum (FBS, Gibco, Australian origin) and incubated in 5% CO_2_ at 37°C. Cells were passaged when the cell growth rate reached approximately 80%.

### Uptake experiment of gelsolin-targeted paclitaxel-loaded poly(lactic-co-glycolic acid) nanoparticles in Hca-F and Hca-P cell lines

Hca-F and Hca-P cells were seeded into 6-well plates (1 × 10^5^ cells/well). The cells were divided into the following four groups: 1) Hca-F cells with PLGA NPs, in which 150 μl of coumarin 6-labeled PLGA NPs was added to Hca-F cells; 2) Hca-F cells with antibody blocking, in which 20 μl of GSN monoclonal antibody was added to Hca-F cells to block the expressed protein on the cell surface for 20 min followed by the addition of 150 μl of coumarin 6-labeled GSN-PLGA NPs; 3) Hca-P cells with GSN-PLGA NPs, in which 150 μl of coumarin 6-labeled GSN-PLGA NPs was added to Hca-P cells; and 4) Hca-F cells with GSN-PLGA NPs, in which 150 μl of coumarin 6-labeled GSN-PLGA NPs was added to Hca-F cells. After incubation for 2 h, cells were washed three times with PBS and then fixed with 4% paraformaldehyde for 15 min. After washing three times, the nuclei in each group were stained with DAPI, and the cells were collected by centrifugation at 8,000 rpm/min in PBS. Cells were visualized using a confocal laser scanning microscope to assess the uptake of NPs in each group.

### Ultrasound imaging of the gelsolin-targeted paclitaxel-loaded poly(lactic-co-glycolic acid) nanoparticles *in vitro*

The capability of GSN-PLGA NPs used as a contrast agent at various concentrations for ultrasound imaging was assessed *in vitro* using the MyLab Twice scanner system with a MI = 0.06. In total, 2 ml of GSN-PLGA NPs at three concentrations (1, 0.5, and 0.25 mg/ml) was added to the agarose gel holes, and 2 ml of degassed water was added to the agarose gel holes as a control.

## Detection and evaluation of GLN-mediated targets *in vivo*

### Establishment of the murine tumor model

Hca-F cells in the logarithmic growth phase were collected and resuspended in PBS. Cells (4 × 10^7^ cells/100 µl) were inoculated in the sole of the left foot in inbred Chinese 615 mice. After 2 weeks, the inoculated tumor cells developed xenografts. Some mice developed metastatic lymph nodes in different regions, including popliteal, inguinal, and iliac artery lymph nodes. All the experimental procedures were approved by the Animal Ethics Committee of Dalian Medical University (201516).

### *In vivo* fluorescence imaging of DiI-labeled gelsolin-targeted paclitaxel-loaded poly(lactic-co-glycolic acid) nanoparticles in the murine tumor model

Four weeks after tumor inoculation, nine mice were selected and randomly divided into experimental, control 1, and control 2 groups. All tumor-bearing mice were anesthetized with 10% chloral hydrate followed by injection of 0.2 ml of DiI-labeled GSN-PLGA NPs, 0.2 ml of DiI-labeled normal saline (NS), and 0.2 ml of DiI-labeled PLGA NPs *via* the tail vein in the experimental, control 1, and control 2 groups, respectively. All mice were placed in a fluorescent imaging black box for imaging. Two hours after injection, all mice were sacrificed by cervical dislocation. The major organs, including the lungs, heart, liver, spleen, and kidneys, as well as implanted tumor tissues and ipsilateral enlarged lymph nodes, were harvested. The isolated organs were placed in the fluorescent imaging dark box according to the normal organ position for fluorescence imaging.

### Tumor growth after targeted treatment with gelsolin-targeted paclitaxel-loaded poly(lactic-co-glycolic acid) nanoparticles

One week after tumor inoculation, 100 mice with tumor masses with diameters of approximately 0.5 cm were selected and randomly divided into five groups (*n* = 20 in each group), namely, the control, PLGA, PTX, PTX-PLGA, and GSN-PTX-PLGA groups, in which the mice were injected with NS, PLGA NPs, PTX solution, PTX-PLGA NPs, and GSN-PTX-PLGA NPs, respectively. When the foot pad was partially tumorigenic, the longitudinal and transverse diameters of the tumor mass were measured every 3 days, and the body mass was weighed. The tumor volume was calculated using the following formula: *V* = (*a* × *b* ^2^)/2, where *a* represents the longitudinal diameter of the tumor and *b* represents the transverse diameter of the tumor. The tumor growth curve of each group was plotted.

Four weeks after treatment, the tumor-bearing mice were sacrificed by cervical dislocation, and the implanted tumor and ipsilateral draining lymph nodes (popliteal, inguinal, and iliac artery lymph nodes) were isolated and fixed in 10% neutral-buffered formalin, paraffin-embedded, and cut into 4 μm sections for hematoxylin and eosin (HE) staining. The potential secondary tumors were examined under a microscope. A secondary tumor in one of these lymph nodes is considered to be lymph node metastasis. The lymph node metastasis rate in each group was calculated.

### Statistical analysis

Statistical analysis was performed using SPSS 22.0 software (International Business Machines, corp., Armonk, NY, United States). Data are expressed as the mean ± standard deviation (SD). Statistical differences for individual groups were compared using the *t*-test. The chi-squared test was used to analyze the proportion of mice with lymphatic metastasis among the different groups. *p* < 0.05 was considered statistically significant.

## Results

### Characterization of gelsolin-targeted paclitaxel-loaded poly(lactic-co-glycolic acid) nanoparticles

**FIGURE 1:**
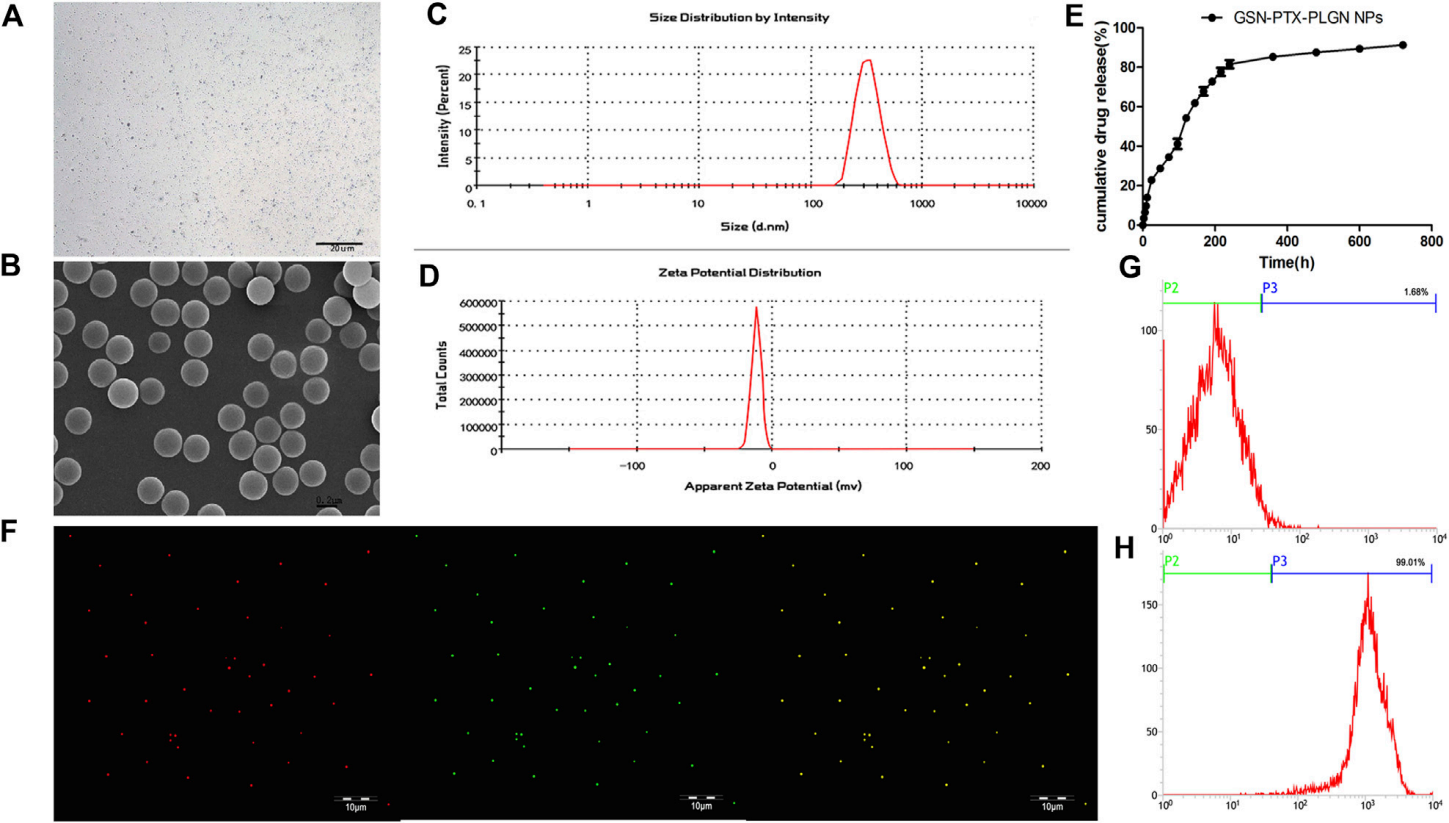
**(A)** Light microscope image of GSN-PTX-PLGA NPs (× 600). **(B)** Scanning electron microscopy image of GSN-PTX-PLGA NPs (× 30,000). **(C)** The size distribution of GSN-PTX-PLGA NPs detected by a laser particle size analyzer. **(D)** The zeta potential of GSN-PTX-PLGA NPs detected by a laser particle size analyzer. **(E)** *In vitro* release curve of GSN-PTX-PLGA NPs. **(F)** Red fluorescent dots were observed after binding with DiI-labeled PLGA NPs. Green fluorescent dots were observed after binding DiI-labeled PTX-PLGA-GSN NPs and the secondary antibody. **(G)** The connection rate of the bound antibody on the surface of PLGA NPs detected by the flow cytometry technique was 1.68%. **(H)** The connection rate of the bound antibody on the surface of GSN-PTX-PLGA NPs detected by the flow cytometry technique was as high as 99.01%.

The GSN-PTX-PLGA NPs prepared in the present study exhibited a smooth and uniform spherical morphology (Figures 1A,B). The diameter of GSN-PTX-PLGA NPs was 328.59 ± 3.82 nm with a zeta potential of −11.46 ± 1.19 mV (Figures 1C,D). When a volume of 20 μl was added during preparation, the encapsulation efficiency of PTX in GSN-PTX-PLGA NPs was 83.1 ± 2.12%, and the drug-loading efficiency was 8.31 ± 0.21%. The release rate of GSN-PTX-PLGA NPs was 22.79% at the 24th h, 85.28% on day 15 and 91.29% on day 30 (Figure 1E).

### Attachment of the gelsolin monoclonal antibody to the surface of gelsolin-targeted paclitaxel-loaded poly(lactic-co-glycolic acid) nanoparticles

DiI-labeled PLGA NPs showed red fluorescent dots under a laser confocal scanning microscope. Green fluorescent dots were observed after binding DiI-labeled PTX-PLGA-GSN NPs and the secondary antibody (Figure 1F). The attachment rate of the bound antibody on the surface of GSN-PTX-PLGA NPs was up to 99.01% (Figure 1H), whereas the attachment rate of the bound antibody on the surface of PLGA NPs was only 1.68% (Figure 1G).

### Gelsolin-targeted paclitaxel-loaded poly(lactic-co-glycolic acid) nanoparticles in Hca-F and Hca-P cells

**FIGURE 2:**
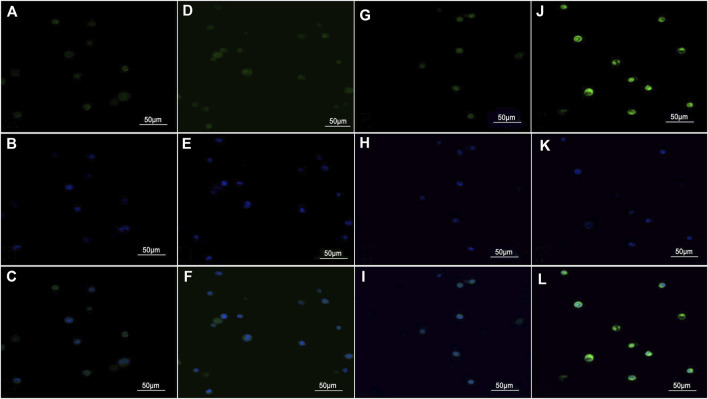
Images were acquired using a laser confocal scanning microscope (×400). **(A,D,G, and J)** Green fluorescence was observed in the cytoplasm of cells after the uptake of the contrast medium. **(B,E,H, and K)** Blue fluorescence was observed in DAPI-stained nuclei. **(C,F,I, and L)** The cytoplasm and the nucleus were merged. **(A–C)** Hca-F cells with PLGA NPs. **(D–F)** Hca-F cells with antibody blocking. **(G–I)** Hca-P cells with GSN-PLGA NPs. **(J–L)** Hca-F cells with GSN-PLGA NPs.

Representative images for the *in vitro* uptake experiments under a laser confocal scanning microscope are shown in Figure 2. Weak green fluorescence was observed in Hca-F cells with PLGA NPs and Hca-F cells with antibody blocking (Figures 2A,D). In addition, the Hca-F cells with GSN-PLGA NPs exhibited stronger green fluorescence than Hca-P cells with GSN-PLGA NPs (Figures 2G,J).

### *In vitro* ultrasound imaging capacity of gelsolin-targeted paclitaxel-loaded poly(lactic-co-glycolic acid) nanoparticles at various concentrations

**FIGURE 3:**
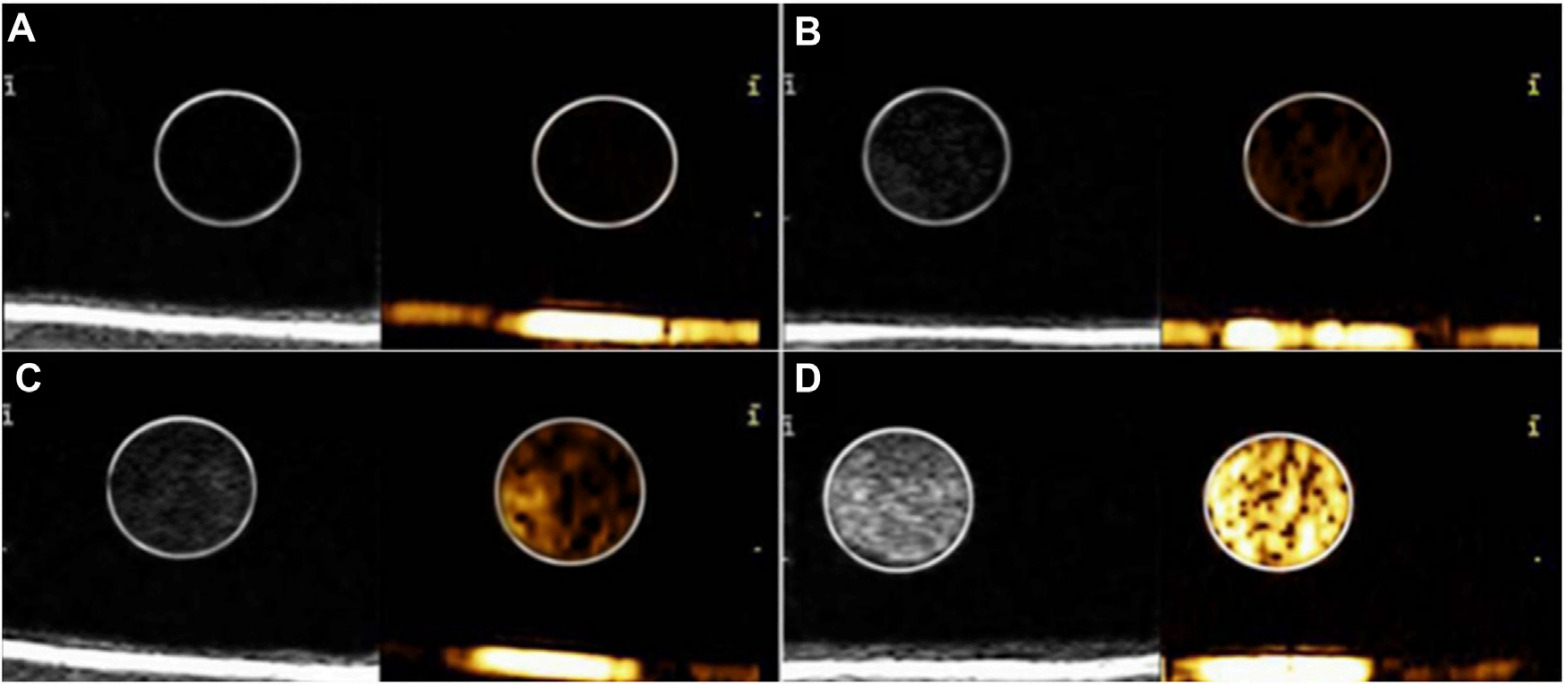
Ultrasound imaging of various concentrations of GSN-PLGA NPs. **(A)** Degassed water as the control group. **(B)** 0.25 mg/ml GSN-PLGA NPs. **(C)** 0.5 mg/ml GSN-PLGA NPs. **(D)** 1 mg/ml GSN-PLGA NPs.

Figure 3 shows that the ultrasound imaging of GSN-PLGA NPs was favorable, with smooth and uniform echogenicity without posterior acoustic attenuation. Importantly, the control was echo-free. In addition, the echo intensity gradually increased with increasing concentrations of GSN-PLGA NPs.

### *In vivo* evaluation of fluorescence imaging of DiI-labeled gelsolin-targeted paclitaxel-loaded poly(lactic-co-glycolic acid) nanoparticles in tumor-bearing mice

**FIGURE 4:**
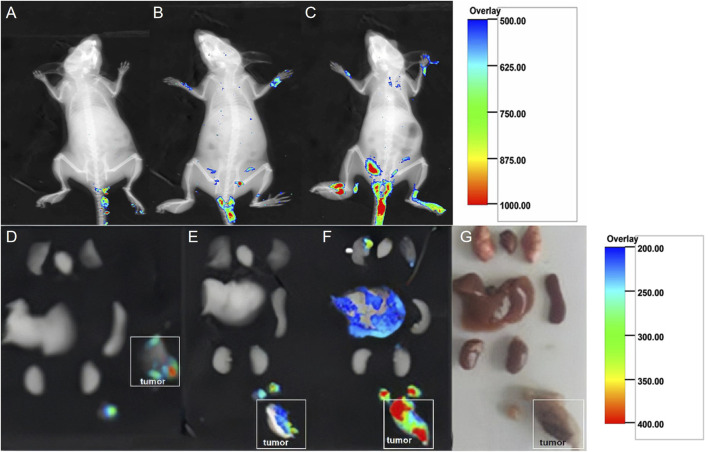
Fluorescence images of living tumor-bearing mice injected with the same dose of fluorescent NPs. **(A)** Control group 1: 0.2 ml of DiI-labeled NS was injected. **(B)** Control group 2: 0.2 ml of DiI-labeled PLGA NPs was injected. **(C)** Experimental group: 0.2 ml of DiI-labeled GSN-PLGA NPs was injected. Fluorescence in the isolated organs, including the lungs, heart, liver, spleen, and kidneys, as well as implanted tumor tissues and ipsilateral enlarged lymph nodes of the mice in order from top to bottom. **(D)** Control group 1: 0.2 ml of DiI-labeled NPs was injected. **(E)** Control group 2: 0.2 ml of DiI-labeled PLGA NPs was injected. **(F)** Experimental group: 0.2 ml of DiI-labeled GSN-PLGA NPs was injected. **(G)** Isolated organs.

The fluorescence imaging of the living tumor-bearing mice injected with the same dose of fluorescent NPs is presented in Figures 4A–C. Compared to the control 1 and control 2 groups (Figures 4A,B), there was significantly stronger fluorescence in the experimental group (Figure 4C). Furthermore, the fluorescence of the isolated organs (Figure 4G), including the lungs, heart, liver, spleen, and kidneys, as well as implanted tumor tissues and ipsilateral enlarged lymph nodes of the mice, is presented in Figures 4D–F. Compared to the control 1 and control 2 groups (Figures 4D,E), the tumor mass and ipsilateral lymph nodes in the experimental group showed more obvious fluorescence aggregation (Figure 4F).

### Tumor growth *in vivo*

There was no significant difference in tumor volume among the tumor-bearing mice before administration (*p* > 0.05). After 1 week, mice received tail vein injections and underwent monitoring. In the NS and PLGA groups, the tumors continued to grow, and limb dysfunction gradually appeared. Three weeks after administration of NS or PLGA, ulceration, suppuration, and gangrene occurred in some mice. The tumor growth state was measured by the size of the tumor. In the PTX group, four mice died after 2 weeks of treatment; they exhibited decreased activity before death. For instance, the skin and mucous membranes were pale, and the skin of two of the dead mice was lighter than that of the other mice. In contrast, mice in the PTX-PLGA and GSN-PTX-PLGA groups were in better condition, and the tumor growth rate was much lower than that in the other two control groups. Moreover, the tumor volume in the GSN-PTX-PLGA group significantly differed from that in the three other groups (*p* < 0.05). The changes in the tumor volume in each group after 4 weeks of drug administration are shown in Supplementary Figure S1; Supplementary Table S1.

### Metastasis rate of lymph nodes in tumor-bearing mice

Four weeks after drug administration, metastasis to the popliteal, inguinal, and iliac artery lymph nodes was observed in all groups. HE staining results of metastasis LN are shown in Supplementary Figure S2. The LN metastasis rates of the NS, PLGA, PTX, PTX-PLGA, and PLGA groups were 80% (16/20), 80% (16/20), 60% (12/20), 60% (12/20), and 25% (5/20), respectively. The lymph node metastasis rate in the GSN-PTX-PLGA group was significantly lower than those in the other groups (*p* < 0.05).

## Discussion

Early identification and intervention of lymphatic metastasis are of great significance in prolonging the survival of patients. In recent years, NPs have been widely used for the diagnosis and targeted treatment of tumors because they are not only good acoustic contrast agents but also efficient vectors for drug delivery (Sennoga et al., 2017; Wang et al., 2018). Furthermore, NPs, with diameters less than 700 nm, penetrate the tumor vascular endothelium and diffuse into the intercellular space of tumors (Lawrie et al., 2000). To date, many new film-forming materials have been developed to prepare NPs. PLGA has been approved by the US Food and Drug Administration (FDA) for clinical applications due to its specific features, including high stability, biodegradability, and ease of chemical modification. NPs prepared with PLGA can be stably combined with specific ligands by chemical bonds (Rapoport et al., 2011). Therefore, PLGA is an ideal material for targeted imaging and treatment. Researchers have reported that the diameters of air-filled PLGA NPs, which are used as a contrast agent for ultrasound imaging, and MTX-loaded PLGA NPs are 370 ± 12 and 477.6 ± 119.7 nm, respectively (Néstor et al., 2011; Zhang et al., 2014). In the present study, we used a modified double-emulsion solvent evaporation technique to successfully prepare GSN-PTX-PLGA NPs with a smooth and uniform spherical morphology and a diameter of 328.59 ± 3.82 nm. Moreover, we confirmed that these NPs can be further used for specific enhanced targeted imaging and treatment of tumors *in vivo* and *in vitro*.

In the last decade, molecular imaging and targeted therapy of tumor neovascularization and tumor cells have been research hotspots. However, studies on the specific development of extravascular molecular targets, such as metastatic lymph nodes and lymphatic vessels, are still limited (Kato et al., 2019). To improve the diagnostic accuracy of metastatic lymph nodes, some researchers have developed new multifunctional contrast agents, which can be used as drug carriers for tumor diagnosis and targeted therapy (Niu et al., 2013). GSN, an important cytoskeletal protein, affects cell morphology and movement by regulating the polymerization and depolymerization of the actin cytoskeleton, thereby playing an important role in cell adhesion, invasion, and migration (Deng et al., 2015; Stock et al., 2015). GSN has been confirmed to be associated with lymphatic metastasis of hepatocarcinoma in mice (Sun et al., 2009; Zhang et al., 2015). The expression of GSN on the membrane of tumor cells is generally accompanied by lymph node metastasis (Qazi et al., 2011). In the present study, GSN was covalently linked to the surface of PLGA-COOH NPs using the carbodiimide technique. Green fluorescent dots of DiI-labeled PTX-PLGA-GSN NPs were observed under a fluorescence microscope after binding with DiI dye and secondary antibody, which showed that GSN was successfully connected to the surface of the contrast agent.

Furthermore, we investigated GSN-mediated targeting capacity *in vivo* and *in vitro*. *In vitro* flow cytometry showed that the amount of antibody bound to the surface of GSN-PTX-PLGA NPs was up to 99.01%, indicating that the prepared GSN-PTX-PLGA NPs had a higher binding rate with the ligand. According to the *in vitro* uptake experiments, weak green fluorescence was observed in Hca-F cells incubated with PLGA NPs and with antibody blocking. In contrast, Hca-F cells incubated with GSN-PLGA NPs exhibited stronger green fluorescence than Hca-P cells with GSN-PLGA NPs. These results further demonstrated a higher expression level of GSN in Hca-F cells than that in Hca-P cells, as found by subcellular proteomics analysis between these two cellular lines in a previous study (Qazi et al., 2011). Therefore, the GSN-PTX-PLGA NPs could effectively bind with the membrane antigen GSN. Moreover, *in vivo* fluorescence imaging showed that DiI-labeled GSN-PLGA NPs accumulated in the implanted tumor tissues and ipsilateral lymph nodes of tumor-bearing mice in both living mouse bodies and isolated organs. The specific binding between GSN-PLGA NPs and Hca-F cells with higher expression of GSN on the cell surface *in vitro* and *in vivo* further indicated that GSN is a suitable target for lymphatic metastasis. GSN-PLGA NPs have capabilities for specific molecular targeting and delivering drugs to tumors and lymph nodes. The imaging of GSN-PLGA NPs was demonstrated to be favorable *in vitro*. However, the imaging of GSN-PLGA NPs *in vivo* was not satisfactory, which may be due to the small particles of the contrast agents, limiting their acoustic reflection and preventing detection by the probe. Another reason may be that the volume of the tumor mass was relatively small in the tumor-bearing mice, which increases the difficulty of detection. The phase-transition material perfluorohexane in the core of GSN-PLGA NPs, which would undergo a liquid-gas phase transition upon activation by US energy, has been considered to improve the imaging enhancement effect in our following studies.

Despite the poor imaging *in vivo*, the targeted drug delivery *in vivo* in this study yielded optimal results. As drug carriers, PLGA NPs could be taken in by endocytosis and have significant advantages, including high drug-loading efficiency, long-term maintenance of blood concentration, favorable controlled release effect, and stability *in vivo* (Bolhassani et al., 2014; Gdowski et al., 2015; Meng et al., 2016). In the present study, the GSN-PTX-PLGA NPs exhibited a higher PTX encapsulation efficiency and drug-loading efficacy. In addition, the lower burst release rate and favorable sustained-release performance indicated that GSN-PTX-PLGA NPs in the bloodstream would decrease the toxicity and side effects of PTX. Regarding the *in vivo* targeting treatment experiments, tumor-bearing mice with PTX-PLGA and GSN-PTX-PLGA lived healthier with a slower tumor growth rate. Furthermore, the tumor volume in the GSN-PTX-PLGA group was significantly different from that in the other groups, and the lymphatic metastasis rate in the GSN-PTX-PLGA group was significantly lower compared to the other groups. These results indicated that GSN-PTX-PLGA NPs more easily pass through the gaps of vascular endothelial cells and arrive at the targeted areas to enrich PTX at the tumor mass due to the guiding effects of GSN.

## Conclusion

In conclusion, GSN-PTX-PLGA NPs were successfully prepared and exhibited a high encapsulation efficiency, drug-loading efficacy. The imaging of GSN-PLGA NPs was satisfactory *in vitro*, and the echo intensity gradually increased with increasing concentrations of GSN-PLGA NPs. After treatment with GSN-PTX-PLGA NPs, there was an obvious decrease in tumor volume and lymph node metastasis in tumor-bearing 615 mice. Overall, GSN-PTX-PLGA NPs have a remarkable targeting capacity *in vivo* and *in vitro*, and they effectively inhibit tumor growth and lymph node metastasis *in vivo*. GSN-PTX-PLGA NPs effectively reduced the toxicity of PTX in mice and increased the drug utilization rate, which contributed to their therapeutic role in suppressing tumor growth and lymph node metastasis. Moreover, although the ultrasound imaging of GSN-PLGA NPs *in vitro* was good, the targeting imaging *in vivo* was unsatisfactory. Therefore, these NPs still require further modifications to improve the imaging quality *in vivo*. Nevertheless, GSN-PTX-PLGA NPs are of great significance in the early identification and treatment of tumors and lymph node metastasis, thereby providing a new perspective for tumor-targeted therapy.

## Data Availability

The raw data supporting the conclusion of this article will be made available by the authors without undue reservation.
