# Supplementary figures and images for: The targeting imaging and treatment capacity of gelsolin-targeted and paclitaxel-loaded PLGA nanoparticles in vitro and in vivo
Source: Front Bioeng Biotechnol. 2022 Oct 20;10:933856. doi: 10.3389/fbioe.2022.933856 (PMC9632342; doi:10.3389/fbioe.2022.933856)

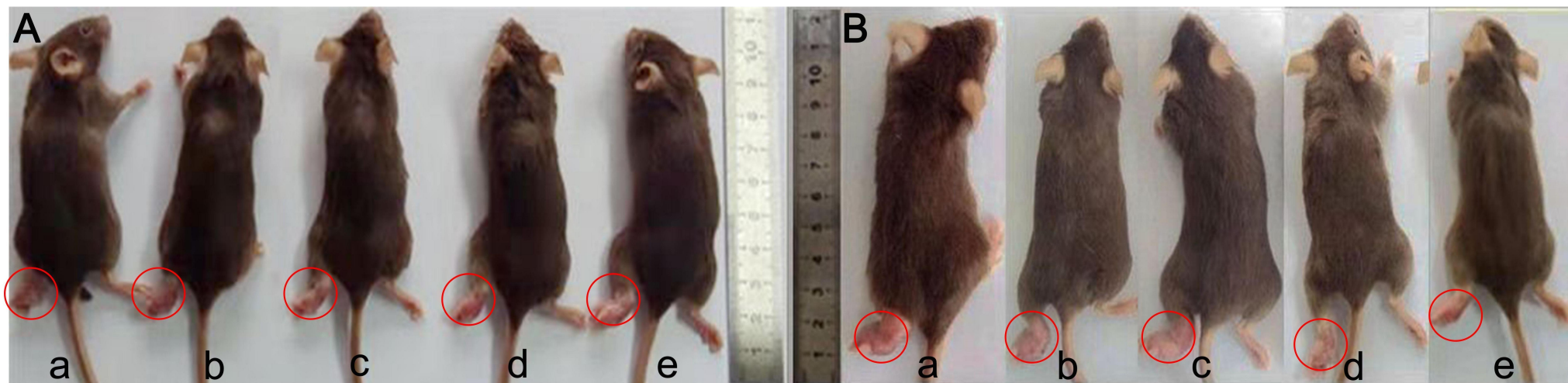

Supplement: Supplementary file 1 [file DataSheet2.PDF]

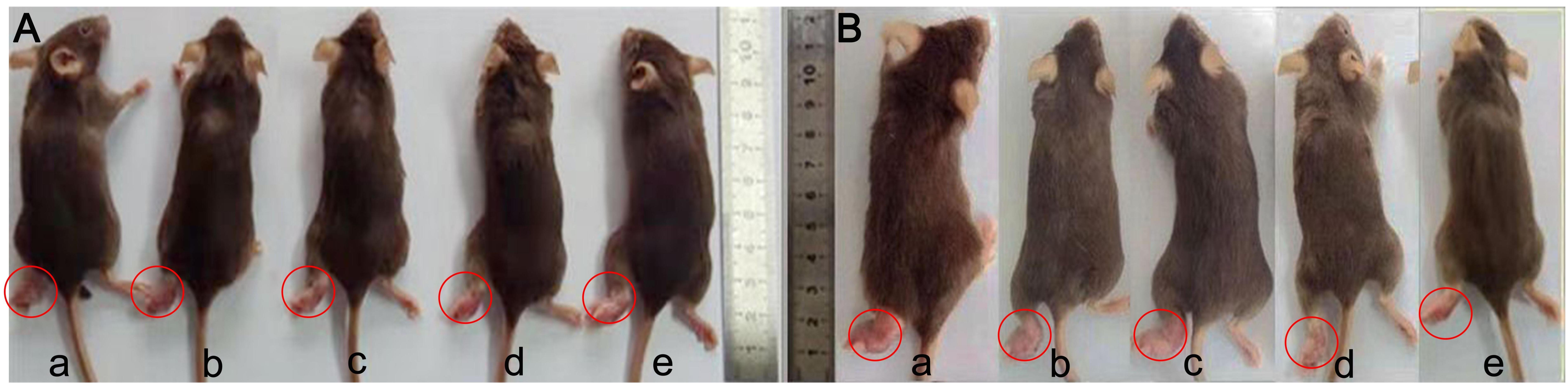

Supplement: Supplementary file 3 [file Image1.JPEG]

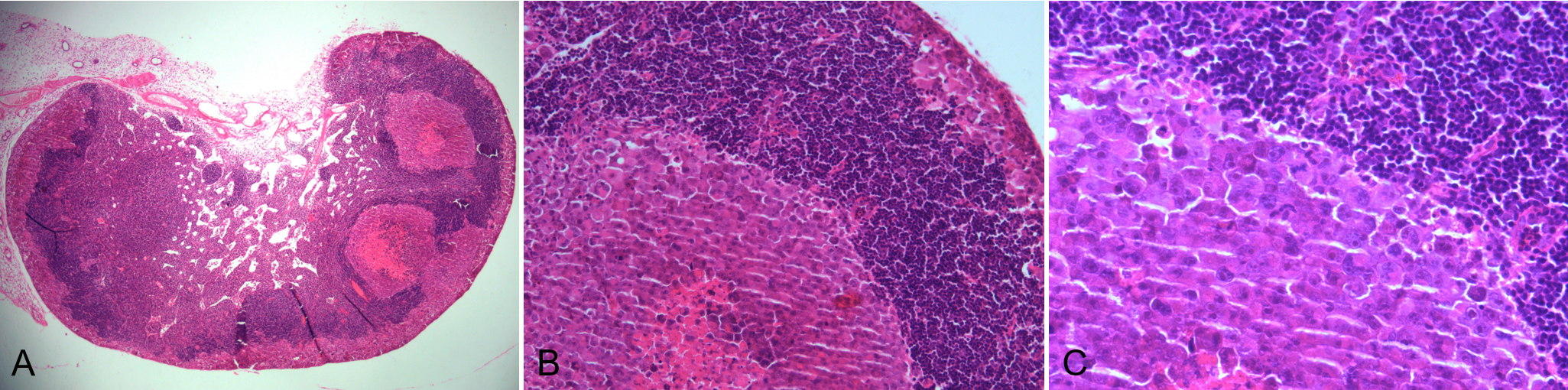

Supplement: Supplementary file 4 [file Image2.TIF]

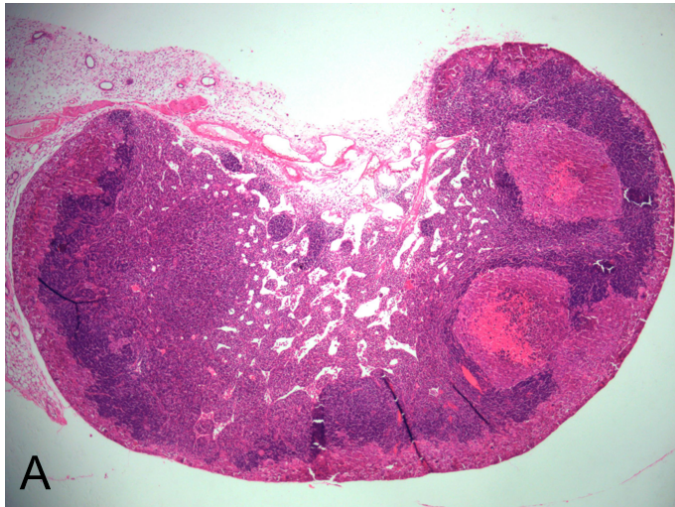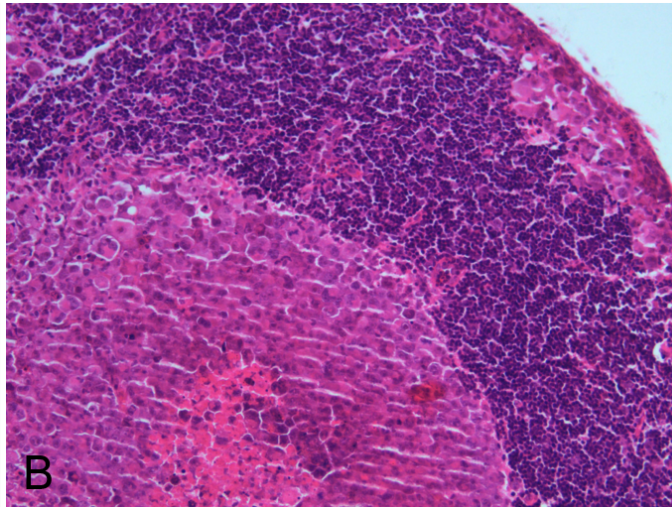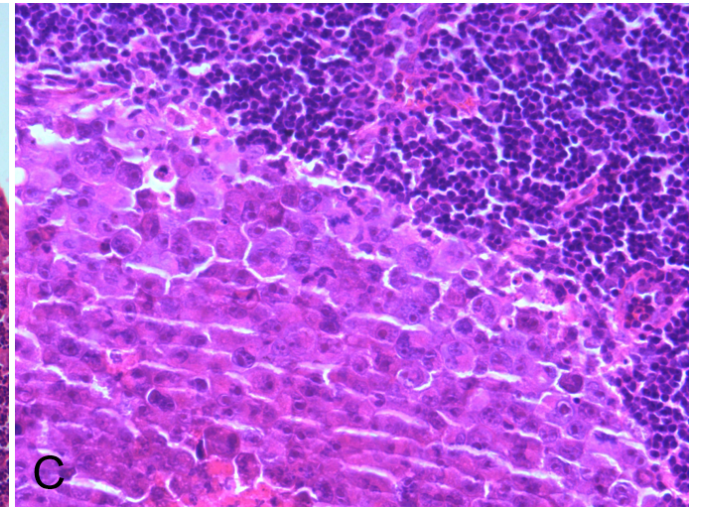

Supplement: Supplementary file 5 [file DataSheet1.PDF]
