# Supplementary material for: The targeting imaging and treatment capacity of gelsolin-targeted and paclitaxel-loaded PLGA nanoparticles in vitro and in vivo
Source: Front Bioeng Biotechnol. 2022 Oct 20;10:933856. doi: 10.3389/fbioe.2022.933856 (PMC9632342; doi:10.3389/fbioe.2022.933856)
Supplement: Supplementary file 2 [file Table1.DOCX]

Supplementary Material

**Supplementary figure 1.** The changes of tumor volume at the left foot pad of mice in each group in the fourth week after drug administration. (A) The tumor volume between tumor-bearing mice before drug administration. (B) The tumor volume between tumor-bearing mice in the fourth week after drug administration. a. NS group; b. PLGA group; c. PTX group; d. PTX-PLGA group; e. GSN-PTX-PLGA group.

**Supplementary figure 2.** HE staining of metastasis LN. (A) Metastasis LN (× 40). (B) Metastasis LN (× 200). (C) Metastasis LN (× 400).

**Supplementary table 1.** The tumor volume in each group in the fourth week after drug administration (V/cm^3^) (means±SD)

| Group | Tumor volume before drug administration | Tumor volume after drug administration |
| --- | --- | --- |
| NS group | 0.058±0.014 | 1.102±0.301 |
| PLGA group | 0.047±0.019 | 0.844±0.248 |
| PTX group | 0.047±0.013 | 0.794±0.438 |
| PTX-PLGA group | 0.059±0.018 | 0.634±0.198* |
| GSN-PTX-PLGA group | 0.047±0.013 | 0.554±0.214* |

*: Compared with NS group, *P*<0.05.
